# Kent and Kleemeier Award Lecture and Presentations

**DOI:** 10.1093/geroni/igab046.1827

**Published:** 2021-12-17

**Authors:** Theresa Harvath

## Abstract

The Donald P. Kent Award lecture will feature an address by the 2020 Kent Award recipient, David Ekerdt, PhD, FGSA, of the University of Kansas. The Kent Award is given annually to a member of The Gerontological Society of America who best exemplifies the highest standards of professional leadership in gerontology through teaching, service, and interpretation of gerontology to the larger society. The Robert W. Kleemeier Award lecture will feature an address by the 2020 Kleemeier Award recipient, Matt Kaeberlein, PhD, FGSA, of the University of Washington. The Kleemeier Award is given annually to a member of The Gerontological Society of America in recognition for outstanding research in the field of gerontology.

